# The Relationship between Visual Impairment and Health-Related Quality of Life in Korean Adults: The Korea National Health and Nutrition Examination Survey (2008–2012)

**DOI:** 10.1371/journal.pone.0132779

**Published:** 2015-07-20

**Authors:** Yuli Park, Jeong Ah Shin, Suk Woo Yang, Hyeon Woo Yim, Hyun Seung Kim, Young-Hoon Park, Epidemiologic Survey Committee of the Korean Ophthalmologic Society

**Affiliations:** 1 Department of Ophthalmology and Visual Science, Yeouido St. Mary's Hospital, College of Medicine, The Catholic University of Korea, Seoul, Korea; 2 Department of Ophthalmology and Visual Science, Seoul St. Mary's Hospital, College of Medicine, The Catholic University of Korea, Seoul, Korea; 3 Department of Preventive Medicine, College of Medicine, The Catholic University of Korea, Seoul, Korea; Karolinska Institutet, ITALY

## Abstract

**Introduction:**

To evaluate health-related quality of life (HRQoL) in Korean adults with visual impairment(VI) using various measures based on a nationally distributed sample.

**Methods:**

Using the Korea National Health and Nutrition Examination Survey (KNHANES, 2008–2012) data, we compared EuroQol five-dimensional questionnaire (EQ-5D) and EQ-visual analogue scale (VAS) scores after adjusting for socio-demographic and psychosocial factors as well as for comorbidities with VI. Logistic regressions were used to elucidate determinants for the lowest quintile HRQoL scales according to VI severity. Uncorrected visual acuity (VA) which implies vision of ordinary life was measured using an international standard vision chart based on Snellen scale.

**Results:**

28,825 participants (sum of weights; 37,562,376) were included in the analysis. The mean EQ-5D and EQ-VAS scores were significantly lower in the VI groups than in the normal vision (defined as VA 20/20-20/25) group based on the better or worse seeing eye (*P*<.0001 and *P*<.0001, respectively). Participants with moderate (VA 20/80-20/160) and severe VI (VA ≤20/200) had higher scores of multivariate-adjusted odd ratios (aORs) for the lowest quintile than did the normal vision group which was particularly evident in the results from EQ-5D, whereas the results of the mild VI (VA 20/32-20/63) group did not identify significant differences from the normal vision group independent of classification according to the better or the worse seeing eye. Conversely, EQ-VAS revealed significantly higher score of multivariate-aORs for the lowest quintile in participants with mild VI either for the better or worse seeing eye.

**Conclusions:**

The severity of VI was definitely associated with impaired HRQoL compared with the normal vision population. The analyses presented here elicited even mild VI could potentially deteriorate the health-related quality of life (or subjective perception of health quality) and therefore, therapeutic approaches should also focus on the subjective perception and better management of health condition.

## Introduction

Visual impairment occurs increasingly frequently as people age. Although a substantial proportion of visual deficit may be reversible by improving spectacle correction or performing a cataract surgery, eventually, preventing complications of chronic disease such a diabetic retinopathy could be considerably important. For example, diabetic retinopathy is the main reason for visual impairment among people of working ages and screening for retinopathy is regarded to be beneficial and cost-effective in preventing visual loss in patients with any chronic disease [1,2]. Dietary restrictions, medications, and chronic disease-associated morbidities seriously deteriorate the quality of life (QoL) of patients with chronic disease [3].

The health-related quality of life (HRQoL) is poorer in patients with chronic disease than in the general population [4,5]. Evaluation of HRQoL is important for evaluating the burden on patients and in selecting treatment methods. HRQoL measurement provides a comprehensive evaluation of the patient’s health status that can provide additional information to laboratory data and subjective symptoms. The value of health states can be estimated by using various measures. These measures include direct valuation methods such as standard gamble (SG) [6], time trade-off (TTO) [7], and the visual analogue scale (VAS) [8] as well as indirect questionnaire-based measures such as the health utilities index mark 3 (HUI-3) [9], the EuroQol five-dimensional questionnaire (EQ-5D) [10], and the 15-D measure [11].

Numerous studies have been carried out to study the prevalence and socioeconomic risk factors for visual impairment across the continents, some of them also investigating the racial and ethnic differences. Although there is growing interest in visual impairment, interest in the HRQoL of patients with visual impairment is relatively low, and no study has focused specifically on the relationship between HRQoL and visual impairment before. The purpose of this study was to investigate the relationship between visual impairment and HRQoL. In this study, we assessed the relationship between HRQoL and visual impairment based on a national health survey in Korea: The fourth and fifth Korea National Health and Nutrition Examination Survey (KNHANES 2008–2012), a national representative survey conducted by the Ministry of Health and Welfare that provides data on vision status and socio-demographic factors.

## Materials and Methods

### Study population

This study was based on the fourth and fifth KNHANES (2008–2012) which was conducted as a national health survey in Korea that used a stratified, multistage, clustered sampling method based on 2005 National Census data to randomly select a population-based sample across 500 national districts to represent the civilian, noninstitutionalized, South Korean population, and sample design and size were estimated properly so that annual survey results could represent the whole population in Korea. The response rate of the KNHANES IV (2007–2009) was 74.3% and that of the KNHANES V (2010–2012) was 76.1%. Raw data from such surveys can be used for the development of health policies and research, except for the survey date and location. Ophthalmologic interviews and examinations involved a population-based random sampling of households across 576 national districts, which were selected by a panel to represent the South Korean population using a stratified, multistage, clustered sampling method based on 2009 National Resident demographics. All members of each selected household were asked to participate in the survey. Surveys prior to KNHANES IV were able to be analyzed and could be considered a national representative sample after 3 years when the survey was completed, but rolling survey sampling methods were applied from KNHANES IV which allowed annual analysis of national representative sample data possible. All examinations and health interviews by trained teams, including ophthalmology residents, were conducted in mobile centers while nutrition survey was carried out in household. This survey was aimed to determine the prevalence of the vision status and common eye diseases in a population-based sample in Korea.

In the present analysis, we limited the study population to adults, aged 19 years or older who participated in all three parts of the survey in addition to ophthalmologic interviews and examinations. We excluded subjects with a history of malignancy because malignancies have obvious deleterious effects on HRQoL. Therefore, our final study population for the analysis included 28,825 participants (sum of weights; 37,562,376).

This survey was reviewed and approved by the Institutional Review Boards (IRBs) of the Korea Centers for Diseases Control and Prevention (IRB #; 2008-04EXP-01-c, 2009-01CON-03-2C, 2010-02CON-21-C, 2011-02CON-06-C and 2012-01EXP-01-2C). This study was reviewed and approved by the IRBs of the Seoul St. Mary’s Hospital College of Medicine the Catholic University of Korea (IRB #KC14RISI0884). This study adhered to the tenets of the Declaration of Helsinki and all participants provided written informed consent after explanation of the purpose and possible consequences of the study.

### Grading of visual impairment on the better or worse seeing eye

Uncorrected visual acuity (UCVA) and/or Best Corrected Distance Visual Acuity (BCVA) were measured at a distance of 4 m using an international standard vision chart based on Snellen scale (Jin’s vision chart, Seoul, Korea; Jin 1997). The participant’s VA was measured in each eye, right followed by left with his or her existing refractive correction if he or she had one. The participant was asked to read numbers, proceeding to the next line if he or she read more than three letters among the five letters correctly. The participant’s VA was defined as the line with the smallest numbers in which he or she read more than three characters accurately. Automated refraction was performed in participants using the autorefractive keratometer (KR8800; Topcon,Tokyo, Japan), followed by retesting of VA after applying pinhole in patients whose VA was below 0.8 based on Snellen chart.

The participants were divided into four groups on the basis of their uncorrected VA which implies vision of ordinary life in the better and worse eye using International Classification of Diseases, Ninth Revision, Clinical Modification / World Health Organization criteria: normal vision (defined as VA 20/10–20/25), mild visual impairment (VA 20/32–20/63), moderate visual impairment (VA 20/80–20/160), and severe visual impairment (VA ≤20/200).

### Independent variables

From the KNHANES (2008–2012) data set, we collected data of various socio-demographic factors, which were obtained through direct interviews using standardized questionnaires:current age (30–39/40–49/50–59/60–69/70 or older), sex (men/women), household income (divided into quartiles), highest educational level reached (elementary school graduates or lower/middle school graduates/high school graduates/university graduates or higher), current job status (employed/unemployed), marital status (single/married/divorce/widow/widower), frequency of alcohol consumption per week (more than or less than twice per week), current smoker (yes/no), level of psychological stress (none, low, moderate, extreme), and continuous depressive symptoms during the past 2 weeks (yes or no). Participants also reported whether they had ever had comorbidities including hypertension, heart diseases (acute myocardial infarction or angina), stroke, arthritis, DM, and chronic renal disease.

### Multiattribute instruments and measurement of HRQoL

Previous analyses suggest that these socio-demographic characteristics may have an independent association with HRQoL and therefore, were considered potential confounders in multi-variable analyses. Those were subsequently equivalised using the McClements equivalence scale and categorized into quintiles because of the non-linear relationship between socio-demographic factors and HRQoL. EQ-5D and EQ-VAS scales were divided into quintiles for logistic regression also because, the distribution of the utility measures was skewed, independent associations were explored using multivariate quintile regression models. The large differences in the lower quintiles may be attributable to differences in the utility weights.

The information on the respondent’s health status was collected by asking the respondent to complete a specific health questionnaire or by conducting an interview following a detailed interview manual. The responses in the questionnaires were then scored by using published tariffs that had been derived by using one of the direct valuation methods. The questionnaire included questions concerning how respondents perceive their own HRQoL in the dimensions of vision, hearing, speech, ambulation, dexterity, emotion, cognition, and pain. The health description resulting from the questionnaire was scored by using a multiattribute scoring function with values elicited from a random sample of the general population using VAS in combination with the SG method. The EuroQoL consists of two parts, the health-status descriptive system (EQ-5D) and a visual analogue scale (EQ-VAS). The EQ-5D records the level of self-reported problems according to five dimensions (mobility, self-care, usual activities, pain/discomfort, and anxiety/depression) [12,13]. Each of the dimensions is assessed based on a single question with three response levels (no problems, some problems, and extreme problems). Using a combination of these items, a single health index score was calculated using the Korea valuation set developed by the Korean Centers for Disease Control and Prevention [14]. Scores on the EQ-5D index range from -0.171 to 1, where 1 indicates no problems in any of the five dimensions, zero indicates death, and negative values indicate a health status worse than death. Next, respondents described their own health status using a VAS ranging from 0 (worst imaginable health) to 100 (best imaginable health) [13].

### Statistical analysis

Basic characteristics of the study population were reported by descriptive statistics and all sample and weight variables were stratified. We used the stratification variables and sampling weights designated by the Korean Centers for Disease Control and Prevention for prevalence calculations, which were based on the sample design for each survey year. Sampling weights were adjusted for non-response according to demographic factors after the surveys were completed. To calculate the weight of KNHANES (2008–2012) in accordance with the guidelines of the 2005 Census of Korea, post-stratification adjustment based on the response rates and extraction rate to include the same distribution of 2005 Korean population in sex and age group of 5-year intervals was performed. Finally, the sum of the weight of KNHANES IV was equal to the Korean population as of 2005. We calculated the weight of KNHANES V in a similar manner in regards to sex and age groups at 5-year intervals. Prevalence estimates for all outcomes were performed for the overall sample and then in age and gender stratified groups. The frequency procedure was performed with clustering as a sampling-district variable, and prevalence was reported with a 95% confidential interval (CI). The descriptive procedure was used to evaluate the distribution of socio-demographic and clinical variables and QoL measurements. The general linear model was used to compare continuous variables with the results of the EQ-5D and EQ-VAS for each chronic disease (diabetes, hypertension, heart diseases, stroke, arthritis, and chronic renal disease) as dependent variables after controlling for age, gender, household income, educational level, current job, marital status, current smoking status, frequency of alcohol consumption, level of stress, self-reported depressive symptom, and other comorbidities. The EQ-5D and EQ-VAS scales were divided into quintiles, and the odd ratios (ORs) of the lowest quintile of these scales in the each visual impairment group were compared with those in the normal vision group after adjusting for age, gender, household income, educational level, current job, marital status, current smoking status, frequency of alcohol consumption, stress level, self-reported depressive symptoms, and other comorbidities (such as hypertension, heart diseases, stroke, arthritis, and chronic renal disease) using the logistic regression procedure. Four models were constructed and the ORs in model 1 were analyzed without adjustment. The ORs in model 2 were adjusted for age and gender. In model 3, the ORs were further adjusted for household income, education level, marital status, percentage of current smokers, frequency of drinking, level of stress and self-reported depressive symptom. In model 4, the ORs were further adjusted for chronic diseases. Data were analyzed using SAS version 9.3 software (Cary, NC, USA) to account for the sample design and sampling weight, which was adjusted for oversampling, nonresponse, and the Korean population. The adjusted odds ratios (aOR) and 95% confidence interval (CI) were calculated. All statistical tests were two-sided at 95% CI and were performed using the SAS version 9.3 software (Cary, NC, USA). *P* values of less than 0.05 were considered statistically significant.

## Results

28,825 participants (sum of weights; 37,562,376) were included in the analysis. Their clinical and socio-demographic characteristics are described in Table 1 (visual impairment in the better eye) and 2 (visual impairment in the worse eye) according to the severity of visual impairment. EQ-5D and EQ-VAS were able to identify significant differences between the different groups of visual impairment in the better eye as well as between the severity levels classified according to the pathological progression or visual impairment in the worse eye. The mean age for participants with mild, moderate and severe visual impairment was significantly higher than that of subjects with normal vision independent of classification according to the better eye or the worse seeing eye (p<0.0001). The education level, marital status, household income, employment state, percentage of current smokers, drinking frequency and level of psychological stress were also significantly different between the four groups. The level of psychological stress was statistically higher in the participants with severe visual impairment in the better eye than those with mild and moderate visual impairment in the better eye. However, interestingly, the participants with normal vision had significantly higher level of psychological stress than did subjects with mild visual impairment independent of classification according to the better eye or the worse seeing eye. Self-reported depressive symptoms in the participants with severe visual impairment in the better eye were significantly higher than those of the participants with normal vision and moderate visual impairment, but the values were similar to those of the participants with mild visual impairment in the better eye. The prevalence of chronic diseases such as hypertension, stroke and chronic renal disease was also significantly higher in the participants with severe visual impairment in the better eye than those of other groups. The prevalence of heart diseases and arthritis was even significantly higher in the participants with mild visual impairment than in the subjects with moderate and severe visual impairment.

**Table 1:** Subject (visual impairment in the better eye) characteristics and EQ-5D/ VAS index scores.

Characteristics	Normal vision (n* = 19,973; N^†^ = 26,967,628)	Mild VI (n* = 5,018; N^†^ = 5,460,419)	Moderate VI (n* = 526; N^†^ = 638,728)	Severe VI (n* = 119; N^†^ = 130,931)	*P* value
Quality of life					
EQ-5D	0.961(0.0008)	0.915(0.0026)	0.907(0.0087)	0.894(0.0155)	< .0001
EQ-VAS	75.586(0.1446)	71.526(0.3678)	72.240(1.0525)	70.949(2.1496)	< .0001
Age, yr	42.83 (0.18)	52.25(0.41)	46.10(1.22)	46.64(2.26)	< .0001
Sex, female %	47.45(0.39)	60.72(0.91)	64.55(2.84)	64.99(5.60)	< .0001
Education, %					
Elementary school	13.58(0.40)	36.78(0.94)	31.39(2.56)	33.09(5.31)	< .0001
Middle school	9.78(0.27)	11.77(0.59)	4.95(1.06)	8.00(2.75)	
High school	41.60(0.55)	31.20(0.97)	42.57(3.08)	36.03(5.76)	
College	35.04(0.63)	20.25(0.82)	21.09(2.42)	22.88(6.28)	
Marital status, %					
Single	22.63(0.58)	18.02(0.87)	33.50(3.04)	34.26(5.44)	< .0001
Married	70.12(0.59)	62.93(0.98)	46.98(2.84)	47.37(5.57)	
Divorce/widow/widower	7.26(0.24)	19.06(0.71)	19.52(2.03)	18.38(3.90)	
House income					
Lowest quartile %	12.17(0.40)	27.62(0.88)	25.40(2.29)	30.86(5.56)	< .0001
Employed, %	67.36(0.46)	55.16(0.95)	52.08(2.76)	44.47(6.19)	< .0001
Heavy alcohol drinker, %	18.46(0.43)	14.57(0.84)	14.26(2.38)	26.30(7.13)	0.0003
Current Smoking, %	27.70(0.42)	20.35(0.76)	22.94(2.52)	21.40(5.20)	< .0001
Stress level,					
Moderate or extreme %	28.66(0.38)	27.08(0.86)	32.46(2.45)	39.35(5.29)	0.017
Depressive mood (> = 2 wks), %	12.47(0.29)	16.19(0.71)	15.02(1.89)	16.14(3.94)	< .0001
BMI, kg/m2	23.67(0.03)	23.74(0.07)	22.85(0.28)	23.21(0.34)	0.009
Waist circumference, cm	80.88(0.12)	81.54(0.20)	78.43(0.67)	80.06(0.95)	< .0001
Triglyceride, mg/dl	133.47(1.05)	135.73(2.39)	111.63(3.90)	137.49(14.29)	< .0001
High blood pressure, %	14.27(0.32)	26.96(0.74)	21.77(2.13)	30.01(4.79)	< .0001
eGFR, mL/min/1.73m^2^	97.24(0.21)	93.63(0.39)	97.18(1.20)	93.49(3.27)	< .0001
Chronic kidney disease, %	1.77(0.11)	5.08(0.34)	5.90(1.11)	9.42(3.47)	< .0001
Stroke, %	1.04(0.08)	2.28(0.21)	2.41(0.66)	2.80(1.33)	< .0001
MI or angina, %	1.56(0.09)	3.16(0.30)	1.79(0.57)	2.96(1.37)	< .0001
Arthritis, %	10.04(0.26)	21.00(0.70)	15.77(1.71)	10.48(2.47)	< .0001
HbA1C	5.76(0.01)	6.05(0.03)	5.95(0.12)	6.06(0.18)	< .0001
DM, %	5.06(0.18)	10.27(10.51)	7.33(1.26)	13.60(3.51)	< .0001

Data are presented as the means ± SE, or % (SE)

* KNHNES participants

^†^ Estimated number of the Korean population aged ≥19.

VI: Visual impairment, EQ-5D: EuroQol five-dimensional questionnaire, EQ-VAS: EQ-visual analogue scale, BMI: Body mass index, eGFR: Estimated glomerular filtration rate, MI: Myocardial infarction, DM: Diabetes mellitus.

The mean EQ-5D and EQ-VAS scores of the participants who had visual impairment in the better eye are presented in Table 1. The mean EQ-5D index score was 0.961 [standard error (SE), 0.0008] for those with normal vision, 0.915 (SE, 0.0026) for those with mild visual impairment, 0.907 (SE, 0.0087) for those with moderate visual impairment and 0.894 (SE, 0.0155) for those with severe visual impairment (all *P*<0.001). The mean EQ-VAS score was 75.586 (SE, 0.1446) for those with normal vision, 71.526 (SE, 0.3678) for those with mild visual impairment 72.240 (SE, 1.0525) for those with moderate visual impairment and 70.949 (SE, 2.1496) for those with severe visual impairment (all *P*<0.001). The mean EQ-5D and EQ-VAS scores of the participants who had visual impairment in the worse eye are presented in Table 2. The mean EQ-5D index score was 0.964 (SE, 0.0007) for those with normal vision, 0.927 (SE, 0.0020) for those with mild visual impairment, 0.909 (SE, 0.0052) for those with moderate visual impairment and 0.901 (SE, 0.0074) for those with severe visual impairment (all *P*<0.001). The mean EQ-VAS score was 75.958 (SE, 0.1496) for those with normal vision, 72.424 (SE, 0.2880) for those with mild visual impairment 72.329 (SE, 0.7486) for those with moderate visual impairment and 68.482 (SE, 0.9636) for those with severe visual impairment (all *P*<0.001). The mean EQ-5D index and EQ-VAS score were significantly higher in the normal eye group than in the group with visual impairment independent of classification according to the better or the worse seeing eye.

**Table 2:** Subject (visual impairment in the worse eye) characteristics and EQ-5D /VAS index scores.

Characteristics	Normal vision (n* = 16,780; N^†^ = 23,250,495)	Mild VI (n* = 6,929; N^†^ = 7,828,527)	Moderate VI (n* = 1,313; N^†^ = 1,482,535)	Severe VI (n* = 668; N^†^ = 708,787)	*P* value
Quality of life					
EQ-5D	0.964(0.0007)	0.927(0.0020)	0.909(0.0052)	0.901(0.0074)	< .0001
EQ-VAS	75.958(0.1496)	72.424(0.2880)	72.329(0.7486)	68.482(0.9636)	< .0001
Age, yr	41.79(0.18)	50.57(0.33)	49.86(0.78)	54.05(1.07)	< .0001
Sex, female %	46.64(0.43)	56.97(0.75)	62.98(1.76)	54.68(2.46)	< .0001
Education, %					
Elementary school	11.54(0.39)	31.62(0.79)	33.93(1.68)	39.94(2.51)	< .0001
Middle school	9.40(0.29)	12.30(0.51)	7.77(0.96)	10.02(1.37)	
High school	42.72(0.60)	32.77(0.81)	36.29(1.86)	33.12(2.56)	
College	36.34(0.67)	23.32(0.74)	22.02(1.55)	16.92(2.22)	
Marital status, %					
Single	23.39(0.63)	18.36(0.74)	23.31(1.86)	18.30(2.29)	< .0001
Married	70.26(0.64)	65.86(0.83)	57.43(1.98)	59.42(2.54)	
Divorce/widow/widower	6.35(0.23)	15.78(0.53)	19.26(1.40)	22.27(1.99)	
House income					
Lowest quartile %	10.98(0.42)	23.01(0.72)	27.56(1.58)	34.03(2.38)	< .0001
Employed, %	68.24(0.49)	58.80(0.81)	53.66(1.88)	49.41(2.56)	< .0001
Heavy alcohol drinker, %	18.63(0.45)	16.23(0.74)	10.69(1.30)	20.49(2.63)	< .0001
Current Smoking, %	28.12(0.46)	22.62(0.69)	20.38(1.52)	23.53(2.35)	< .0001
Stress level,					
Moderate or extreme %	29.00(0.42)	26.92(0.71)	29.04(1.56)	28.47(2.19)	0.074
Depressive mood (> = 2 wks), %	12.22(0.31)	15.01(0.56)	16.17(1.27)	16.43(1.82)	< .0001
BMI, kg/m2	23.64(0.04)	23.80(0.06)	23.41(0.15)	23.28(0.18)	0.005
Waist circumference, cm	80.70(0.12)	81.79(0.18)	80.17(0.39)	81.25(0.50)	< .0001
Triglyceride, mg/dl	133.34(1.14)	134.35(2.18)	123.08(3.13)	148.04(6.36)	0.0004
High blood pressure, %	12.82(0.32)	24.99(0.66)	24.75(1.42)	30.16(2.14)	< .0001
eGFR, mL/min/1.73m^2^	97.64(0.22)	94.42(0.32)	94.45(0.76)	92.01(1.09)	< .0001
Chronic kidney disease, %	1.56(0.11)	4.05(0.26)	5.33(0.68)	7.34(1.27)	< .0001
Stroke, %	0.87(0.07)	2.05(0.16)	2.36(0.45)	4.00(0.82)	< .0001
MI or angina, %	1.36(0.09)	2.93(0.25)	2.27(0.45)	4.58(0.86)	< .0001
Arthritis, %	8.93(0.26)	18.95(0.59)	20.56(1.29)	16.14(1.49)	< .0001
HbA1C	5.73(0.02)	5.97(0.03)	6.12(0.07)	6.11(0.08)	< .0001
DM, %	4.44(0.18)	9.20(0.41)	10.36(1.01)	12.66(1.48)	< .0001

Data are presented as the means ± SE, or % (SE)

* KNHNES participants

^†^ Estimated number of the Korean population aged ≥19.

VI: Visual impairment, EQ-5D: EuroQol five-dimensional questionnaire, EQ-VAS: EQ-visual analogue scale, BMI: Body mass index, eGFR: Estimated glomerular filtration rate, MI: Myocardial infarction, DM: Diabetes mellitus.

The ORs for the lowest quintile of the EQ-5D index and EQ-VAS score according to visual impairment grading of the better eye are presented in Table 3. In model 4, after adjusting for other factors including comorbidities, the OR for the lowest quintile of the EQ-5D index in the mild visual impairment group was 1.08 (95% CI, 0.93 to 1.25), 1.67 (95% CI, 1.10 to 2.53) in the moderate visual impairment group, and 3.39 (95% CI, 1.46 to 7.84) in the severe visual impairment group. In model 4, after adjusting for other factors including comorbidities, the OR for the lowest quintile of the EQ-VAS score in the mild visual impairment group was 1.24 (95% CI, 1.10 to 1.40), 1.29 (95% CI, 0.91 to 1.81) in the moderate visual impairment group, and 1.64 (95% CI, 0.97 to 2.79) in the severe visual impairment group. The ORs for the lowest quintile of the EQ-5D index and EQ-VAS score according to visual impairment grading of the worse eye are shown in Table 4. In model 4, after adjusting for other factors including comorbidities, the OR for the lowest quintile of the EQ-5D index in the mild visual impairment group was 1.11 (95% CI, 0.98 to 1.26), 1.42 (95% CI, 1.11 to 1.81) in the moderate visual impairment group, and 1.55 (95% CI, 1.06 to 2.23) in the severe visual impairment group. In model 4, after adjusting for other factors including comorbidities, the OR for the lowest quintile of the EQ-VAS score in the mild visual impairment group was 1.14 (95% CI, 1.02 to 1.28), 1.17 (95% CI, 0.92 to 1.49) in the moderate visual impairment group, and 1.71 (95% CI, 1.29 to 2.26) in the severe visual impairment group. Participants with moderate and severe visual impairment had higher scores of multivariate-adjusted odd ratios for the lowest quintile than did the normal vision group, which was particularly evident in the results from EQ-5D whereas the results of the mild visual impairment group did not identified significant differences from normal vision group independent of classification according to the better or the worse seeing eye. Conversely, EQ-VAS revealed significantly higher scores of multivariate-adjusted odd ratios for the lowest quintile in participants with mild visual impairment either for the better or worse seeing eye.

**Table 3:** Multivariate-adjusted odd ratios for the lowest quintile of the EQ-5D and EQ-visual analogue scale (VAS) scores for visual impairment groups in the better eye.

EQ-5D							
	OR (95% CI)		*P* value		*P* value		*P* value
Model	Normal vision	Mild VI		Moderate VI		Severe VI	
Model 1	1	2.38(2.18–2.60)	< .0001	2.42(1.91–3.06)	< .0001	3.64(2.29–5.79)	< .0001
Model 2	1	1.36(1.23–1.50)	< .0001	1.82(1.37–2.42)	< .0001	3.02(1.66–5.49)	0.0003
Model 3	1	1.09(0.94–1.26)	0.2389	1.63(1.08–2.47)	0.0194	3.23(1.38–7.54)	0.0069
Model 4	1	1.08(0.93–1.25)	0.3101	1.67(1.10–2.53)	0.0151	3.39(1.46–7.84)	0.0044
EQ-VAS							
	OR (95% CI)		*P* value		*P* value		*P* value
Model	Normal vision	Mild VI		Moderate VI		Severe VI	
Model 1	1	1.84(1.69–2.01)	< .0001	1.47(1.14–1.90)	0.003	2.23(1.44–3.47)	0.0004
Model 2	1	1.41(1.29–1.54)	< .0001	1.26(0.97–1.64)	0.0791	2.00(1.27–3.14)	0.0027
Model 3	1	1.25(1.11–1.41)	0.0002	1.29(0.91–1.82)	0.1488	1.65(0.98–2.77)	0.0601
Model 4	1	1.24(1.10–1.40)	0.0004	1.29(0.91–1.81)	0.1476	1.64(0.97–2.79)	0.0647

Model 1, no adjustments; Model 2, adjusted for age and sex; Model 3, adjusted for age, sex, household income, education level, marital status, occupational status, smoking, alcohol, stress and presence of depression; Model 4, adjusted for age, sex, household income, education level, marital status, occupational status, smoking, alcohol, stress, presence of depression other chronic diseases (diabetes, hypertension, heart disease, stroke, arthritis, and chronic kidney disease).

OR, odds ratio; CI, confidence interval.

VI: Visual impairment, EQ-5D: EuroQol five-dimensional questionnaire, EQ-VAS: EQ-visual analogue scale.

**Table 4:** Multivariate-adjusted odd ratios for the lowest quintile of the EQ-5D and EQ-visual analogue scale (VAS) scores for visual impairment groups in the worse eye.

EQ-5D							
	OR (95% CI)		*P* value		*P* value		*P* value
Model	Normal vision	Mild VI		Moderate VI		Severe VI	
Model 1	1	2.19(2.02–2.37)	< .0001	2.81(2.42–3.26)	< .0001	3.39(2.75–4.18)	< .0001
Model 2	1	1.31(1.19–1.43)	< .0001	1.70(1.45–1.99)	< .0001	1.85(1.43–2.38)	< .0001
Model 3	1	1.13(1.00–1.28)	0.0533	1.40(1.10–1.78)	0.0064	1.43(0.98–2.08)	0.0604
Model 4	1	1.11(0.98–1.26)	0.0955	1.42(1.11–1.81)	0.0050	1.55(1.06–2.23)	0.0241
EQ-VAS							
	OR (95% CI)		*P* value		*P* value		*P* value
Model	Normal vision	Mild VI		Moderate VI		Severe VI	
Model 1	1	1.65(1.53–1.79)	< .0001	1.72(1.46–2.03)	< .0001	2.62(2.15–3.19)	< .0001
Model 2	1	1.30(1.19–1.41)	< .0001	1.33(1.13–1.57)	0.0006	1.96(1.59–2.40)	< .0001
Model 3	1	1.16(1.03–1.29)	0.0105	1.18(0.93–1.50)	0.1740	1.66(1.26–2.19)	0.0003
Model 4	1	1.14(1.02–1.28)	0.0199	1.17(0.92–1.49)	0.1887	1.71(1.29–2.26)	0.0002

Model 1, no adjustments; Model 2, adjusted for age and sex; Model 3, adjusted for age, sex, household income, education level, marital status, occupational status, smoking, alcohol, stress and presence of depression; Model 4, adjusted for age, sex, household income, education level, marital status, occupational status, smoking, alcohol, stress, presence of depression other chronic diseases (diabetes, hypertension, heart disease, stroke, arthritis, and chronic kidney disease).

OR, odds ratio; CI, confidence interval.

VI: Visual impairment, EQ-5D: EuroQol five-dimensional questionnaire, EQ-VAS: EQ-visual analogue scale.

## Discussion

In this study, we estimated HRQoL with visual impairment severity and investigated whether differences in HRQoL exist between severity levels of the vision. HRQoL can be affected by various factors and of these factors, we examined HbA1c level, depressive symptom, psychological stress, and comorbidities such as chronic kidney disease, high blood pressure, stroke, myocardial infarction or angina and arthritis. The mean EQ-5D and EQ-VAS scores were significantly lower in the visual impairment groups than in the normal vision group based on the better or worse seeing eye (*P* < .0001 and *P* < .0001, respectively). Participants with moderate and severe visual impairment had higher scores of multivariate-adjusted odd ratios for the lowest quintile than did the normal vision group which was particularly evident in the results from EQ-5D whereas the results of the mild visual impairment group did not identify significant differences from the normal vision group independent of classification according to the better eye or the worse seeing eye. On the other hand, EQ-VAS revealed significantly higher scores of multivariate-adjusted odd ratios for the lowest quintile in participants with mild visual impairment either for the better or the worse seeing eye. Although the moderate and severe visual impairment groups had higher scores of multivariate-adjusted odd ratios for the lowest quintile than did the normal vision group which was particularly evident in the results from the EQ-VAS, independent of classification according to the better eye or the worse eye, the difference was not statistically significant. When participants were classified according to visual impairment in the better eye, EQ-5D and EQ-VAS resulted in a score ordering that is somewhat contradicted our hypothesized expectations. Contrary to our expectations, the scores did not always follow the progression of the disease and the severity of visual impairment.

Numerous demographic and psychosocial factors such as age, gender, depressive symptom, and psychological stress influenced QoL [15,16]. Actually, OR was largely attenuated in models 2, 3, and 4 (from 3.64 to 1.08) indicating that differences in age, income, education and complications of chronic disease play a large role in the observed difference in HRQoL. However, we also found that EQ-5D scores remained low in participants with mild visual impairment compared with those with moderate or severe visual impairment in the better or the worse eye even after adjusting for many socio-demographic and psychosocial factors and comorbidities. The complications of chronic diseases might lower HRQoL compared with the values in the general population regardless of other related factors [16]. The causal relationship between socio-demographic factors and visual impairment, whether the socio-demographic disparities are the cause or the consequence of visual impairment, is not clear. However, the results of our study revealed significant disparities in socio-demographic factors between the visually impaired and the normal subjects. People of older age, low educational status, the unemployed, and living without a spouse were more likely to have visual impairment. In particular, participants of the older age and the absence of a spouse were unable to undergo vision evaluation (refusal of examination, anophthalmia, or did not know the letters), reflective of poorer eye sight. These results can be explained in relevance to the factors related to visual impairment analyzed above and indicate that participants who are unable to undergo evaluation of visual acuity or who have low visual acuity area more likely to be socio-demographically of a lower class [17].

We measured the health status by EQ-5D and calculated the EQ-5D score to investigate the relationship between EQ-5D and EQ-VAS scores and visual impairment severity. In this research, we predicted that HRQoL would decrease according to the severity of visual impairment, partially could be explained by the existence of complications from chronic disease, and included demographic parameters of the participants. A significant difference in EQ-5D and EQ-VAS score was detected in visual severity grading. In addition, visual impairment is also considered to contribute to a lower HRQoL. It has been generally expected that participants with decreased visual acuity of the better eye probably would have lower EQ-5D score than those with decreased visual acuity of the worse eye and also according to visual impairment severity, it is expected that the participants with severe visual impairment would probably have lower EQ-5D score than those with mild visual impairment. However, contrary to our hypothesis, our study showed different results that no significant differences were revealed independent of classification according to the better eye or the worse eye and HRQoL did not precisely worsened as the order of visual impairment severity. A possible reason for this result is that participants would adapt to their own health state. This corresponds well with our results for the EQ-5D, where the participants in the moderate and severe visual impairment group exhibited higher scores of multivariate-adjusted odd ratios for the lowest quintile than normal vision independent of classification according to the better eye or the worse eye. Adaption could perhaps also explain why participants with mild visual impairment reported significantly higher EQ-VAS scores than did those with normal vision whereas the participants with moderate and severe visual impairment did not show statistically significant higher EQ-VAS scores than those of normal vision group. The participants with moderate and severe visual impairment may adapt to their ordinary life well enough not to feel any discomfort for long periods of the chronic phase, but those who perhaps acutely lost visual ability might feel severe discomfort of ordinary life even with mild impairment of vision. Subjective symptoms and EQ-5D health status were strongly related, which was reflected in the HRQoL. Most importantly, a significant relationship between the number of subjective symptoms, including decreased visual acuity and EQ-5D scores, was observed. The EQ-5D clearly decreased if subjective symptoms were present and reflected the characteristic of each of the 5D. These findings suggest the value of assessing visual impairment by the EQ-5D because it would allow the comprehensive evaluation of the participants’ health conditions and add another dimension to the subjective symptoms and laboratory data. We demonstrated the relationship between participants’ grades of visual impairment and the EQ-5D, and that the EQ-5D score was affected by the number of symptoms. The EQ-5D score may also be able to detect subtle aspects that cannot be gathered by asking participants about their symptoms. Nevertheless, the EQ-5D, which is less sensitive than disease-specific scales, should be used in combination with the disease-specific scale for clinical evaluations. The ED-5D scores alone appear to be more suitable for cost-utility analyses. We measured HRQoL using the EQ-5D and the EQ-VAS, one of the preference-based measures among HRQoL instruments that enable the calculation of the utility value.

In addition, the questionnaires of the indirect methods, such as the EQ-5D, limit participants’ descriptions of their health states to specific dimensions. Whereas the EQ-5D score measures HRQoL indirectly from the five domains, the VAS measures it directly by the participants’ subjective feelings. Therefore, the VAS more clearly demonstrates the severity of the disease that the participant himself/herself experiences. It has been argued that potentially positive aspects that would be captured with the direct methods might be missed by the indirect estimations [17]. This also could explain the results which the EQ-5D only revealed the moderate and severe visual impairment group had higher scores of multivariate-adjusted odd ratios for the lowest quintile than did the normal vision group, whereas the EQ-VAS revealed significantly higher scores of multivariate-adjusted odd ratios for the lowest quintile in participants with mild visual impairment either for the better or the worse seeing eye. Another possible explanation could be that no participant reported full health with EQ-VAS but the difference could also be due to the different methodologies. Our results demonstrated that the severity of visual impairment had a positive correlation with EQ-5D scores in the better and worse eye. There are some preference-based instruments, such as the Rosser Index, the 15D, the Quality of Well-Being scale, the Health Utility Index (HUI) versions II and III, and the SF-6D that are based on the generic SF-36 health survey [18]. It has been suggested in some reports that the SF-36-based SF-6D is more sensitive than the EQ-5D in healthy people and for the detection of small health changes, particularly at the extremes of the scale [19,20]. However, in the ADVANCE trial, algorithms based on survey instruments including more comprehensive aspects of HRQoL did not appear to measure greater variations in utility than those based on simpler instruments, such as the EQ-5D. Moreover, the EQ-5D is easier to use and less time-consuming [21]. Therefore, the EQ-5D has some advantages, particularly for large population studies such as the KNHANES. The strength of our study was that these data were obtained from a nationwide population with a high response rate and therefore provided representative information on the Korean population.

Nevertheless, several study limitations should be considered. First, although the visual impairment caused by chronic disease such as diabetic retinopathy was closely related to individual HRQoL level [3], we did not assess all possible causes of visual impairment because the KNHANES is not a survey only for a specific disease and does not provide all data related to that specific disease. Further studies on HRQoL in participants with each comorbidity are needed. Second, we could not measure HRQoL using tools other than the EQ-5D and EQ-VAS. Studies are needed that use disease-specific QoL tools to assess the association between diseases and HRQoL. Third, the questionnaires were administered in the same order to all participants. This could potentially have biased our comparison because of carryover effects or fatigue. Administration order, however, has been found in various previous studies to have no or only a marginal effect on HRQoL measurements [22–24]. Fourth, we did not use any visual support for the EQ-VAS. Because some of the participants with severe visual impairment in the study could have produced a severe bias if, using any form of visual aid so, those participants were unable to use any such aid. In terms of visual impairment, we analyzed it by the uncorrected visual acuity, which implies the vision of each participant’s ordinary life and this exactly reveals the factors that are associated with HRQoL better than the corrected visual acuity. In addition, EQ-VAS exercises administered by telephone have previously been found to give similar results as do face-to-face interviews [25–27].

In this study, we examined the relationship between visual impairment severity and HRQoL that could be used to populate decision models, and many of the models [28–31], that have been developed have been based on the pathological progression in the worse eye. Although HRQoL may mostly be affected by the participants’ ability to see, worries about the disease may also affect their HRQoL. Considering that mild visual impairment can eventually progress to become more severe, it is important to intensively screen such participants at risk and provide treatment before the condition becomes irreversible. Not only would this be beneficial for the participant, but it would also save public health care budgets by intervening as secondary prevention before the condition requires tertiary prevention.

In conclusion, we found that visual impairment severity was clearly associated with impaired HRQoL compared with a population of normal vision. The analyses presented here elicited even mild visual impairment could potentially deteriorate the health-related quality of life, therefore, therapeutic approaches should focus more on the subjective perception of health by participants with visual impairment and further studies on HRQoL are needed for Korean adults with visual impairment.
